# The Use of Proton Radiation in the Management of Adenoid Cystic Carcinoma

**DOI:** 10.1016/j.ijpt.2025.101206

**Published:** 2025-10-08

**Authors:** Irini Yacoub, Achraf Shamseddine, Daniel Kallini, Nader Mohamed, Kaveh Zakeri, Yao Yu, Linda Chen, Daphna Gelblum, Sean McBride, Nadeem Riaz, Eric Sherman, Richard J. Wong, Marc Cohen, Loren Scott Michel, Ian Ganly, Lara Dunn, Alan Ho, Zhigang Zhang, Nicolas Toumbacaris, Nancy Y. Lee

**Affiliations:** 1 Department of Radiation Oncology, New York Proton Center, New York, NY; 2 Department of Radiation Oncology, Memorial Sloan Kettering Cancer Center, New York, NY; 3 Rowan University School of Medicine, Glassboro, NJ; 4 Department of Radiation Oncology, New York University, New York, NY; 5 Department of Medical Oncology, Memorial Sloan Kettering Cancer Center, New York, NY; 6 Department of Epidemiology and Biostatistics, Memorial Sloan Kettering Cancer Center, New York, NY; 7 Department of Surgery, Memorial Sloan Kettering Cancer Center, New York, NY

**Keywords:** Proton, Adenoid cystic, Recurrence, Survival, Adjuvant, Definitive

## Abstract

**Purpose/Objective(s):**

Adenoid cystic carcinoma (ACC) of the head and neck is a rare malignancy with a prolonged but infiltrative course, marked by perineural invasion and high risk of local recurrence and distant metastases. The standard of care for head/neck ACC is surgery and postoperative radiation. Definitive radiation is reserved for those with definitive disease. Given the advantage of proton beam to tailor its dose around the tumor while sparing critical tissues, we sought to report our proton experience in the treatment of head/neck ACC.

**Materials/Methods:**

We retrospectively analyzed 106 patients treated with definitive or adjuvant proton therapy for ACC from 2012 to 2023. All patients were staged with computed tomography (CT), magnetic resonance imaging (MRI), and/or positron emission tomography (PET) and were evaluated by a multidisciplinary team. Radiation doses were ≥66 Gy(RBE) adjuvantly and ≥70 Gy(RBE) definitively. Patients were treated using either uniform scanning or pencil beam scanning (PBS) proton therapy. Primary endpoints included overall survival (OS), progression-free survival (PFS), and locoregional recurrence (LRR), assessed using Kaplan-Meier and cumulative incidence methods.

**Results:**

Of 106 patients, 76 were treated postoperatively and 30 definitively. The 5-year OS was 75% overall, 86% for the adjuvant group, and 45% for the definitive group. Five-year PFS was 47% overall, 54% (adjuvant) vs 29% (definitive). Locoregional control at 5 years was 89% overall, with 93% for adjuvant vs 74% for definitive treatment. Toxicities were generally mild, with acute grade ≤2 dermatitis (53%) and mucositis (33%) most common. Chronic toxicities included xerostomia (38%) and temporal lobe necrosis (4%).

**Conclusion:**

Proton therapy for ACC of the head and neck yields excellent locoregional control, particularly in the adjuvant setting, with manageable toxicity. For patients with unresectable disease or those seeking organ preservation, definitive proton therapy remains a viable, durable treatment option. These findings support proton therapy as a preferred modality in head and neck ACC.

## Introduction

Adenoid cystic carcinoma (ACC) of the head and neck involving primarily either the major or minor salivary glands (mSG) accounts for 1% of all head and neck malignancies and is the second most common malignant salivary gland tumor.^1^ The median age of presentation is in the fourth decade, with ages as young as 17 years reported.^1^, ^2^ These tumors are marked by a prolonged but highly infiltrative clinical course, a high rate of perineural invasion, frequent local recurrences, and have a predilection for distant metastases.^3^

The standard treatment for ACC of the HN requires complete surgical resection followed by postoperative radiation therapy, given the high risk of local relapse. Adjuvant chemotherapy may be of benefit for patients who have poor pathologic features such as high-grade tumors with >30% solid component. The results of phase II randomized study (RTOG 1008) for salivary gland tumors, including ACC with high-risk features will definitively answer whether there is benefit with the addition of weekly cisplatin.

Given the proclivity for perineural involvement with intracranial extension, complete pathologic resection may not always be feasible.^4^ Further, patients with unresectable disease and those medically inoperable or refused surgery, definitive radiation preferably with chemotherapy is one treatment option. Retrospective studies have reported a wide range of locoregional control ranging from 36% to 93%, depending on the radiation technique,^5^ with non-surgical treatment, with one study using the National Cancer Database demonstrating a survival benefit.^6^ Given the relatively younger age of patients presenting with this malignancy, its propensity to track along cranial nerves and occur in highly sensitive areas of the head and neck, such as the skull base, and the abundance of sensitive and vital structures within the head and neck, it is imperative to minimize treatment side effects whilst ensuring effective tumor control in these patients.

Proton therapy offers a unique advantage due to the ability to define its range using the Bragg peak, thereby virtually sparing toxicity to nearby normal structure. Studies utilizing proton radiation for head and neck ACC have demonstrated good outcomes with low rates of toxicity but are limited in sample size.^7^ The purpose of this paper is to report the largest study to our knowledge on the outcomes at a single institution using proton radiation for head and neck ACC.

## Methods

A retrospective analysis of all patients who received definitive proton therapy treated from November 2012 to December 2023 for ACC with proton radiation was conducted for this study. This encompassed patients with both resected and unresected disease, with or without metastases, upon initial diagnosis (patients receiving re-irradiation in the recurrent setting will be published separately). All patients were evaluated by a head and neck surgeon, medical oncologist, and radiation oncologist. Imaging used to stage patients included computed tomography, magnetic resonance imaging, or positron emission tomography, and also used when necessary for treatment planning. This research was authorized by the Memorial Sloan Kettering Cancer Center (MSKCC) and New York Proton Center (NYPC) Institutional Review Boards, and a waiver for informed consent was granted due to the retrospective nature of the study.

Proton therapy was delivered as an alternative to intensity-modulated radiation therapy off trial or if tumor size made it difficult to safely deliver intensity-modulated radiation therapy while meeting normal tissue constraints. Patients did not receive proton radiation therapy (RT) if it was their preference, if insurance did not approve it, or if there were logistical issues making daily visits to the proton treatment centers difficult, as they are located at different campuses from the MSKCC campus. Patients were treated both at Procure (Somerset, New Jersey) and NYPC (New York, New York). Proton therapy at Procure was delivered using a Proteus 235 system (Ion Beam Applications, Louvain-la-Neuve, Belgium). At NYPC, it was delivered using a Varian ProBeam system (Varian Medical Systems, Palo Alto, California). Radiation was delivered with either uniform scanning or pencil beam scanning (PBS) using 3 to 5 fields. Multi-field optimization was reserved for more complicated cases (typically larger or more locally advanced tumors) to maximize conformality and reduce dose to nearby organs at risk, and single-field uniform dose was the most commonly utilized, otherwise. The use of intensity modulated proton therapy began in December 2015. For both uniform scanning and PBS plans, setup variations were accounted for by compensator smear or simulated isocenter shift variations of between 3 and 5 mm, respectively. The clinical target volume was expanded to a planning treatment volume typically 3 mm to account for intrafraction patient motion and interfraction setup error, but 1 mm was allowed for disease near critical structures, especially when tracing nerves to the skull base or intracranially, in cases of perineural invasion. Robustness tests, including range uncertainty of ±3.5%, were performed to ensure that clinical target volume V95 > 95%. A generic relative biological effectiveness of 1.1 was used for dose evaluation. All patients were aligned by using orthogonal radiographs on a 6-degrees-of-freedom couch. At NYPC, all patients are treated with PBS protons. In this article, the use of intensity modulated proton therapy will be referring to any PBS plan (both single-field uniform dose and multi-field optimization).

All patients were evaluated by a head and neck surgeon to evaluate for resection. In the adjuvant setting, a dose of 60 GyE or higher depending on the extent of resection was given. Patients treated definitively were given at-least 70 GyE to the gross tumor volume as defined by imaging studies and clinical examination. Nodal irradiation was given in the setting of pathologically involved nodes.

This retrospective study received IRB approval, MSKCC IRB 16-1648. Patient, tumor, and treatment characteristics were recorded and analyzed with descriptive statistics. Radiation treatment records and oncologic outcomes were manually abstracted from electronic medical records, using a uniform data abstraction form. Primary endpoints were locoregional control, progression-free survival (PFS), and overall survival (OS). Patient demographics and treatment characteristics were summarized using medians and interquartile ranges for continuous variables and counts and percentages for categorical variables. OS was defined as time from end of RT to date of last follow up or death. PFS was defined as time from end of RT to date of last follow-up or death. PFS was defined as the time from the end of RT to the date of local, regional, distant recurrence, or death, or last follow-up. Local or regional failures were confirmed by both imaging studies and tissue biopsies. Kaplan-Meier analysis was used to estimate OS and PFS and log-rank tests were utilized to compare survival curves. Locoregional recurrence (LRR) was defined as the time from the end of RT to the date of local or regional failure or last follow-up. Death without locoregional failure was the competing event. Patients were censored at the date of last follow-up. LRR was analyzed using cumulative incidence curves and Gray’s test was utilized to test for differences in cumulative incidence curves. Univariable Fine-Gray models were utilized to assess the associations of dose with LRR. All statistical tests were two-sided and a *P* value less than 0.05 was considered statistically significant. Statistical analyses were conducted using R (version 4.4.1, R Core Development Team, Vienna, Austria).

## Results

This analysis included 106 patients treated with proton therapy for ACC. Patient characteristics are reported in Table 1. Seventy-six patients were treated in the adjuvant setting, and 30 in the definitive setting. The most common sites for ACC were parotid (*N* = 22, 20%), submandibular (*N* = 17, 16%), and oral cavity (*N* = 19, 18%). Fifteen patients (14%) had nodal disease, and *N* = 14 (13%) patients had metastatic disease at diagnosis. Fifty-five patients (51%) received systemic therapy, with cisplatin-based regimens being the most common. Thirty-two patients (30%) had base of skull extension. Two patients had recurrent but not previously radiated ACC. Thirty-two patients did not undergo resection, either due to a morbid surgery or patient declining a disfiguring procedure. The median radiation dose for postoperative patients is 66 GyE, and 70 GyE for definitive patients. Two patients received Quadshot regimen (1 cycle, and 3 cycles) for definitive radiation.

**Table 1:** Characteristics of patients treated in the primary setting.

Characteristic	Patients (*N* = 106)	
	Adjuvant (*N* = 76)	Definitive (*N* = 30)
*Age, y*		
Median (IQR)	59 (45, 68)	54 (40, 69)
*Tumor stage*		
T1	23 (30)	0 (0)
T2	15 (20)	2 (7)
T3	11 (14)	2 (7)
T4	27 (36)	26 (86)
*Nodal Stage*		
N0	66 (87)	26 (86)
N1	5 (7)	2 (7)
N2	3 (4)	2 (7)
N3	2(2)	0 (0)
*Metastatic Stage*		
M0	73 (96)	20 (67)
M1	3 (4)	10 (33)
*Margin Status*		
R0	9 (12)	--
Close (<5 mm)	18 (23)	--
R1	46 (61)	--
R2	3 (4)	--
*Perineural Invasion*		
Yes	54 (71)	--
No	22 (29)	--
*Chemotherapy*		
Yes	30 (39)	25 (83)
No	46 (61)	5 (17)
*Type of Chemotherapy*		
Cisplatin	22 (73.3)	21 (84)
Carboplatin	1 (3.3)	1 (4)
Doxorubicin	7 (23.3)	3 (12)
*Base of Skull extension*		
Yes	12 (16)	20 (67)
No	64 (84)	10 (33)
*Primary Site*		
Parotid	21 (28)	1 (3)
Submandibular	17 (22)	--
Sublingual	3 (4)	--
Sinonasal (Nasal cavity, maxillary sinus, ethmoid sinus, sphenoid sinus)	9 (12)	6 (20)
Base of Skull/Cavernous sinus/Clivus	1 (1)	7 (23)
Nasopharynx	4 (5)	7 (23)
Oropharynx (BOT, soft palate)	1 (1)	1 (3)
Oral Cavity (Hard palate, FOM, buccal, lip)	15 (20)	4 (3)
Orbit/Lacrimal gland	1 (1)	1 (3)
Larynx/Trachea	1 (1)	3 (10)
External auditory canal	3 (4)	--
*Recurrent*		
Yes	2 (3)	0 (0)
No	74 (97)	30 (100)
*Radiation dose (median, IQR), Gy RBE*	6600 (IQR 6006, 6618)	7000 (IQR 7000, 7073)

## Overall-survival

**Figure 1:**
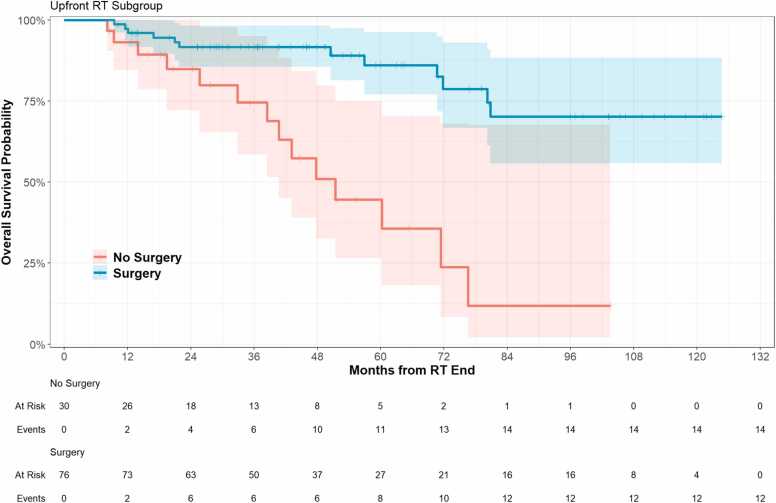
Overall survival outcomes by surgical status.

OS probability is reported in Table 2 and shown in Figure 1. OS probability at 12-, 24-, and 48-months is 96% (93%, 100%), 90% (84%, 96%), and 81% (73%, 90%), respectively. For patients treated post-operatively, OS at 12- 24- and 48-months is 97% (94%, 100%), 92% (85%, 98%), and 92% (85%, 98%) whereas it is 93% (85%, 100%), 85% (72%, 100%) and 51% (33%, 80%) for those treated with definitive radiation (Figure 1).

**Table 2:** Overall survival. Progression-free survival and locoregional control outcomes.

Overall survival probability (95% CI)	All patients				
12 mo	96% (93%, 100%)				
24 mo	90% (84%, 96%)				
36 mo	87% (81%, 94%)				
48 mo	81% (73%, 90%)				
60 mo	75% (65%, 86%)				
96 mo	55% (42%, 72%)				
*Overall Survival Probability by group (95% CI)*	*Adjuvant (N* = *76)*		*Definitive (N = 30)*		
12 mo	97% (94%, 100%)		93% (85%, 100%)		
24 mo	92% (85%, 98%)		85% (72%, 100%)		
36 mo	92% (85%, 98%)		75% (58%, 95%)		
48 mo	92% (85%, 98%)		51% (33%, 80%)		
60 mo	86% (77%, 96%)		45% (27%, 75%)		
96 mo	70% (56%, 88%)		12% (2.1%, 68%)		
*Overall Survival Probability by stage (95% CI)*	*T1*	*T2*	*T3*		*T4*
12 mo	100% (100%, 100%)	100% (100%, 100%)	92% (79%, 100%)		94% (89%, 100%)
24 mo	100% (100%, 100%)	100% (100%, 100%)	92% (79%, 100%)		81% (70%, 93%)
36 mo	100% (100%, 100%)	100% (100%, 100%)	92% (79%, 100%)		75% (64%, 89%)
48 mo	100% (100%, 100%)	100% (100%, 100%)	83% (64%, 100%)		66% (52%, 83%)
60 mo	92% (77%, 100%)	100% (100%, 100%)	66% (40%, 100%)		62% (48%, 80%)
96 mo	92% (77%, 100%)	80% (52%, 100%)	66% (40%, 100%)		24% (10%, 57%)
*Locoregional Recurrence (95% CI)*	*All Patients*				
12 mo	2.8% (0.75%, 7.3%)				
24 mo	5.0% (1.8%, 10%)				
36 mo	7.4% (3.2%, 14%)				
48 mo	9.2% (4.1%, 17%)				
60 mo	11% (5.3%, 20%)				
96 mo	14% (6.7%, 25%)				
*Locoregional Recurrence (95% CI)*	*Adjuvant (N = 76)*			*Definitive (N = 30)*	
12 mo	1.3% (0.11%, 6.4%)			6.9% (1.2%, 20%)	
24 mo	2.7% (0.50%, 8.4%)			12% (2.7%, 28%)	
36 mo	4.2% (1.1%, 11%)			17% (4.9%, 36%)	
48 mo	6.7% (2.0%, 15%)			17% (4.9%, 36%)	
60 mo	6.7% (2.0%, 15%)			26% (7.5%, 49%)	
96 mo	10% (3.3%, 22%)			26% (7.5%, 49%)	
*Progression Free Survival (95% CI)*	*All Patients*				
12 mo	83% (76%, 90%)				
24 mo	73% (65%, 83%)				
36 mo	63% (54%, 74%)				
48 mo	54% (44%, 66%)				
60 mo	47% (37%, 61%)				
96 mo	32% (21%, 49%)				
*Progression Free Survival (95% CI)*	*Adjuvant (N = 76)*			*Definitive (N = 30)*	
12 mo	91% (84%, 98%)			63% (48%, 83%)	
24 mo	85% (77%, 94%)			42% (26%, 66%)	
36 mo	73% (63%, 85%)			36% (21%, 62%)	
48 mo	60% (48%, 75%)			36% (21%, 62%)	
60 mo	54% (42%, 70%)			29% (15%, 58%)	
96 mo	38% (25%, 59%)			15% (3.1%, 68%)

OS differed by T, N, and M-stage. At 48-months, OS for T1, T2, T3, and T4 disease was 100% (95% CI 100%, 100%), 100% (95% CI 100%, 100%), 83% (95% CI 64%, 100%), and 66% (95% CI 52%, 83%), respectively. At 48-months, OS for node positive patients was 61% (95% CI 39%, 95%), compared to 84% (95% CI 76%, 93%) for node negative patients. 48-month OS for non-metastatic patients was 85% (95% CI 77%, 94%), versus 54% (95% CI 31%, 96%) for patients with metastases.

## Progression-free survival

**Figure 2:**
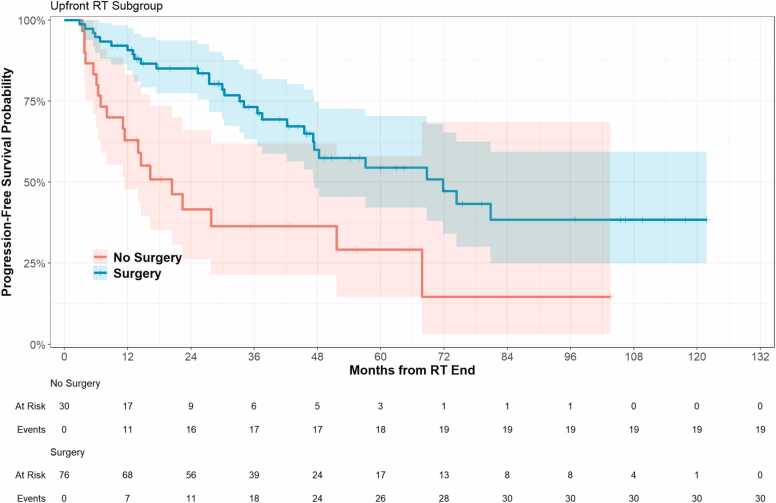
Progress free survival outcomes by surgical status.

PFS probability is reported in Table 2 and shown in Figure 2. PFS for the entire cohort at 12, 24, and 48-months was 83% (95% CI 76%, 90%), 73% (95% CI 65%, 83%), and 54% (95% CI 44%, 66%), respectively. For patients who received surgery, PFS at 12-, 24-, and 48-months was 91% (95% CI 84%, 98%), 85% (95% CI 77%, 94%), and 60% (48%, 75%) compared to 63% (95% CI 48%, 83%) and 42% (95% CI 26%, 66%), and 36% (21%, 62%) for patients who did not undergo surgery (Figure 2).

PFS by T stage at 48-months was 78% (60%, 100%), 73% (50%, 100%), 91% (75%, 100%), and 62% (48%, 79%) for T1, T2, T3, and T4 stage, respectively. Similarly, 48-month PFS for patients with any nodal disease was 58% (35%, 96%) compared to 74% (64%, 85%) for those with N0 disease. For patients with metastatic disease at diagnosis, 48-month PFS was 14% (95% CI 4%, 52%), and 60% (95% CI 49%, 73%) for those with M0 disease.

## Locoregional recurrence

**Figure 3:**
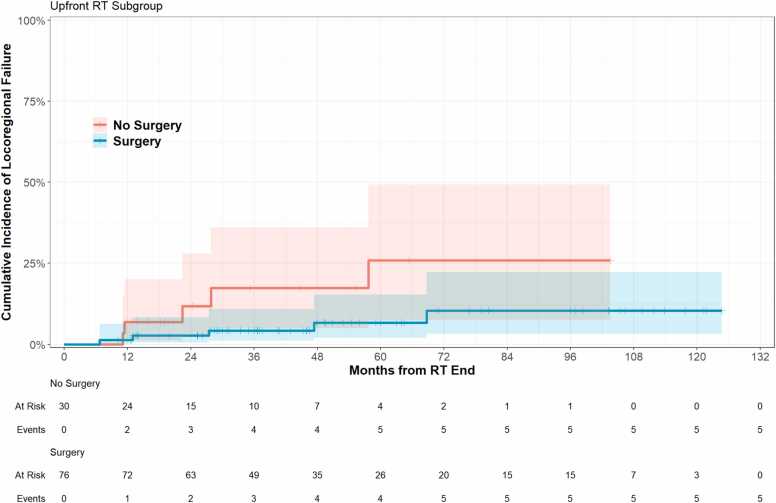
Cumulative incidence of locoregional failure by surgical status.

Cumulative incidence of LRR is reported in Table 2 and shown in Figure 3. LRR for the entire cohort at 12-, 24-, and 48-months was 2.9% (95% CI 0.77%, 7.5%), 5.0% (95% CI 1.8%, 11%), and 9.2% (95% 4.1%, 17%), respectively. At 48 months, LRR was 6.7% (95% CI 2%, 15%) for patients who underwent resection, compared to 17% (95% CI 4.9%, 36%) for patients who did not.

## Chemotherapy

There was a larger proportion of patients with advanced T stage who received chemotherapy (41/53 patients (77%) with T4 disease received chemotherapy). Similarly, of the 15 patients with nodal disease, 10, 67% received chemotherapy. Additionally, more patients with metastatic disease received chemotherapy (10/13 patients (77%) with metastases). Of the 30 patients who did not receive surgery, 25 (83%) received chemotherapy. The HR was 3.15(95% CI 1.36, 7.31, *P* = 0.007) for patients who received systemic therapy compared to no systemic therapy, and no difference in cumulative incidence of LRR between the treatment groups (HR 2.54, (95% CI 0.65, 9.93), Gray’s test *P* = 0.066).

## Toxicity

Common acute toxicities were all grade 1 or 2, and included xerostomia (*N* = 43, 40%), trismus (*N* = 23, 21%), dysphagia (*N* = 29, 27%), odynophagia (*N* = 25, 23%), dysgeusia (*N* = 27, 25%), dermatitis (*N* = 57, 53%), and mucositis (*N* = 36, 33%). Chronic toxicities included xerostomia (*N* = 41, 38%), trismus (*N* = 27, 25%), dysgeusia (*N* = 10, 9%), hearing changes (*N* = 11, 10%), osteoradionecrosis (*N* = 3, 3%), and temporal lobe necrosis (*N* = 4, 4%). Of the 3 ORN cases, one patient required extensive bone debridement and an obturator. The remaining two were asymptomatic and required only antibiotics. Of the four TLN cases, one patient was symptomatic with seizures requiring Keppra, and the other 3 were asymptomatic and not requiring any intervention.

## Discussion

ACC are tumors of the salivary glands, characterized by a prolonged but highly infiltrative clinical course, a high rate of perineural invasion, frequent local recurrences, and a predilection for presenting or recurring metastatically.^3^ T stage, age, the presence of nodal disease, perineural and lymphovascular space invasion, as well as close or positive margins are all risk factors for recurrent disease,^8^ with patients developing local recurrence having a significantly lower OS.^8^ Thus, local control is imperative in the management of this disease.

Although surgical resection is the mainstay of treatment, complete surgical resection with negative margins is often difficult to achieve due to the proximity to sensitive locations in the head and neck. As such, the addition of postoperative radiation is almost always indicated to improve locoregional control. Surgery alone is associated with low recurrence-free survival rates of 63% and 25% at 5 and 10 years, respectively,^9^ and high local failure rates at 30% at 5 years.^10^ A systematic review of the benefit of the addition of postoperative radiation therapy demonstrated a local control benefit, although without a survival benefit.^11^

Due to the sensitive location of critical structures in the head and neck, the use of particle therapy has been explored, owing to their ability to spare tissue beyond the Braggpeak. In a study of 25 patients with ACC (20 treated adjuvantly) with a median dose of 72 GyE, 2-year local control rates were as high as 95%, and 86% for recurrent disease. In our study of 106, locoregional control was excellent for the entire cohort (89% at 5-years), but was even better for patients who were able to undergo resection, with locoregional control at 5-years of 93%.

Radiation alone has generally been demonstrated to be inferior to surgery and radiation. In a study by Mendenhall et al, 5- and 10-year local control rates with the use of definitive photon radiation alone was 56% and 43%, compared to 94% and 91% with surgery and radiation.^12^ Consequently, radiation alone is hardly recommended for ACC. However, for ACC of critical structures, organ preservation is critical not only for maintaining the physiological and psychological health of patients but also for improving overall quality of life.

Literature on the use of definitive particle therapy has shown better outcomes compared to the use of definitive photon radiation. Akbaba et al also reported on 8 patients who received organ-preserving chemoradiation (CRT) alone for laryngeal ACC, with only one patient recurring and requiring a total laryngectomy 11 years after RT.^13^ Takagi et al reported on 80 patients with ACC of the head and neck treated with proton or carbon ion radiotherapy alone, without chemotherapy, and demonstrated a respectable 5-year local control rates of 68% for inoperable disease.^14^ In our study, our patients treated with definitive proton radiation had a 5-year LRC of 75%, and this remained at 75% at 8 years. These findings demonstrate that organ preservation with definitive RT or CRT is a viable and durable option for patients who either cannot have surgery or may want to avoid surgery to maintain a good quality of life. The majority of patients who did not undergo resection had ACC of mSG. Our high locoregional control rates in these patients suggests a role for proton RT in the setting of ACC of mSG due to non-resectability. These patients may also benefit from systemic therapy, although clear indications for the use of chemotherapy in ACC have not yet been elucidated.

Given ACC is a rare tumor, recommendations on the use of systemic therapy are undefined. Some of the largest studies evaluating the use of chemotherapy are limited to the recurrent or metastatic setting.^15^ An et al conducted one of the largest retrospective analyses evaluating a survival benefit added by adjuvant chemotherapy in 114 patients with resected ACC. The addition of chemotherapy to radiation did not yield a survival improvement in the entire population, but for patients with positive surgical margins, CRT was associated with improvement OS compared to RT alone, as well as in patients with four of more lymph nodes.^16^ We found use of chemotherapy to be associated with OS but not LRR. However, it is important to note that this is likely confounded by the use of chemotherapy in patients who did not undergo surgery, had higher T stage, nodal disease, or had metastatic disease.

Limitations of this study include its retrospective and non-randomized nature. However, it is one of the largest studies published to date evaluating the role of proton beam radiation in the management of both resected and unresected ACC. Another limitation is the lack of a comparison group. However, this is a study on both oncologic outcomes and toxicity data of patients treated at a high-volume academic tertiary care center, and these promising findings may be used to apply to patients treated at other centers.

## Conclusion

Proton therapy offers distinct advantages for patients with ACC. One of the key benefits is the utilization of the Bragg peak phenomenon, allowing for dose escalation,which is particularly crucial in the head and neck region, where ACC commonly invades critical structures due to its tendency for perineural invasion. The favorable locoregional control rates achieved with proton therapy make it a highly viable option for patients with ACC, especially those in whom surgical intervention may cause significant morbidity and poor quality of life. Given these promising findings, proton therapy may lead to a paradigm shift in the management of ACC through prioritizing organ preservation and limited treatment-related morbidity. However, longer follow-up will be needed to determine the durability of these excellent LRC rates.

## CRediT authorship contribution statement

Irini Yacoub: Investigation, Methodology, Writing – original draft, Writing – review & editing. Achraf Shamseddine: Writing – review & editing. Daniel Kallini: Data curation, Writing – review & editing. Nader Mohamed: Data curation, Writing – review & editing. Kaveh Zakeri: Writing – review & editing. Yao Yu: Writing – review & editing. Linda Chen: Writing – review & editing. Daphna Gelblum: Writing – review & editing. Sean McBride: Writing – review & editing. Nadeem Riaz: Writing – review & editing. Eric Sherman: Writing – review & editing. Richard J. Wong: Writing – review & editing. Marc Cohen: Writing – review & editing. Loren Scott Michel: Writing – review & editing. Ian Ganly: Writing – review & editing. Lara Dunn: Writing – review & editing. Alan Ho: Writing – review & editing. Zhigang Zhang: Statistical analysis, Writing – review & editing. Nicolas Toumbacaris: Statistical analysis, Writing – review & editing. Nancy Y. Lee: Conceptualization, Methodology, Supervision, Writing – review & editing.

## Declaration of Competing Interest

The authors declare the following financial interests/personal relationships, which may be considered as potential competing interests: Given their role as associate editor of the International Journal of Particle Therapy, Dr. Nancy Lee had no involvement in the peer review of this article and has no access to information regarding its peer review. Full responsibility for the editorial process for this article was delegated to another journal editor.

## Acknowledgments

Nancy Lee, MD, FASTRO, is an Associate Editor of the International Journal of Particle Therapy.
